# Influence of Tyrosine Kinase Inhibitors on Hypertension and Nephrotoxicity in Metastatic Renal Cell Cancer Patients

**DOI:** 10.3390/ijms17122073

**Published:** 2016-12-09

**Authors:** Aleksandra Semeniuk-Wojtaś, Arkadiusz Lubas, Rafał Stec, Cezary Szczylik, Stanisław Niemczyk

**Affiliations:** Military Institute of Medicine Szaserów, 128 Street, 04-141 Warsaw, Poland; asemeniuk@wim.mil.pl (A.S.-W.); rstec@wim.mil.pl (R.S.); cszczylik@wim.mil.pl (C.S.); sniemczyk@wim.mil.pl (S.N.)

**Keywords:** nephrotoxicity, tyrosine kinase inhibitor, renal cell carcinoma, hypertension

## Abstract

Renal cell carcinoma (RCC) is one of the most common kidney malignancies. An upgraded comprehension of the molecular biology implicated in the development of cancer has stimulated an increase in research and development of innovative antitumor therapies. The aim of the study was to analyze the medical literature for hypertension and renal toxicities as the adverse events of the vascular endothelial growth factor (VEGF) signaling pathway inhibitor (anti-VEGF) therapy. Relevant studies were identified in PubMed and ClinicalTrials.gov databases. Eligible studies were phase III and IV prospective clinical trials, meta-analyses and retrospective studies that had described events of hypertension or nephrotoxicity for patients who received anti-VEGF therapy. A total of 48 studies were included in the systematic review. The incidence of any grade hypertension ranged from 17% to 49.6%. Proteinuria and increased creatinine levels were ascertained in 8% to 73% and 5% to 65.6% of patients, respectively. These adverse events are most often mild in severity but may sometimes lead to treatment discontinuation. Nephrotoxicity and hypertension are related to multiple mechanisms; however, one of the main disturbances in those patients is VEGF inhibition. There is a significant risk of developing hypertension and renal dysfunction among patients receiving anti-VEGF treatment; however, there is also some evidence that these side effects may be used as biomarkers of response to antiangiogenic agents.

## 1. Introduction

Renal cell carcinoma (RCC) is one of the most frequent kidney malignancies and the development of RCC metastases is a major cause of tumor-associated deaths. The morbidity of RCC has increased and RCC is now one of the most common cancers; however, the therapeutic opportunities for metastatic renal cell carcinoma (mRCC) has been rapidly developing [1]. An upgraded comprehension of the molecular biology implicated in the progression of cancer has stimulated an increase in both research and development of innovative antitumor therapies. Inhibition of the vascular endothelial growth factor (VEGF) pathways has been one of the most successful directions to date in rearranging scientific discoveries to clinical benefit for the treatment of malignancies.

VEGF is one of the family members of proteins expressed in multiple tissues and cells that play a key role in angiogenesis during embryogenesis, wound healing and tumor growth. Specific cells that express VEGF include progenitor endothelial cells, endothelial cells (EC), podocytes, fibroblasts, macrophages, and certain tumor types. The proangiogenic effect of VEGF is mediated primarily through receptor tyrosine kinases (RTKs) defined as vascular endothelial growth factor receptor 2 (VEGFR-2) on endothelial cells. Upon ligation and autophosphorylation of VEGFR-2, numerous intracellular signaling pathways are activated and mediate the effects of VEGF on endothelial cell survival, proliferation, and migration [2]. The agents administered in the treatment of mRCC include anti-VEGF monoclonal antibody (mAb) (bevacizumab), the mammalian target of rapamycin (mTOR) inhibitors (everolimus and temsirolimus) and the most commonly used, the tyrosine-kinase inhibitors (TKIs), such as sunitinib, pazopanib, sorafenib, and axitinib. Due to the fact that these agents target a number of kinases, they are not selective. For example, sunitinib inhibits VEGFR-1, VEGFR-2, platelet-derived growth factor (PDGFR), stem cell factor receptor (c-KIT), fms-like tyrosine kinase-3 (Flt3), colony stimulating factor receptor type 1 and the receptor encoded by the ret proto-oncogene (RET). Axitinib, which is the most selective drug, interacts with VEGFR-1, VEGFR-2, and VEGFR-3 [3]. All of these anti-VEGF drugs share similar side effects, the most frequent of which are gastrointestinal disturbance, skin toxicity, fatigue and hypertension. Renal side effects are also frequently reported, but their exact rate is not known. The mechanisms that trigger the renal toxicities related to TKIs are complex. Targeted agents can cause damage to the microvasculature, glomerulus, tubules, and interstitium, yielding clinical outcomes. Clinically, the manifestations of kidney injuries reported ranged from an asymptomatic proteinuria to renal failure. This article will review this class effect in terms of frequency and clinical implications, as well as discussing potential mechanisms for these toxicities.

## 2. Results

**Figure 1:**
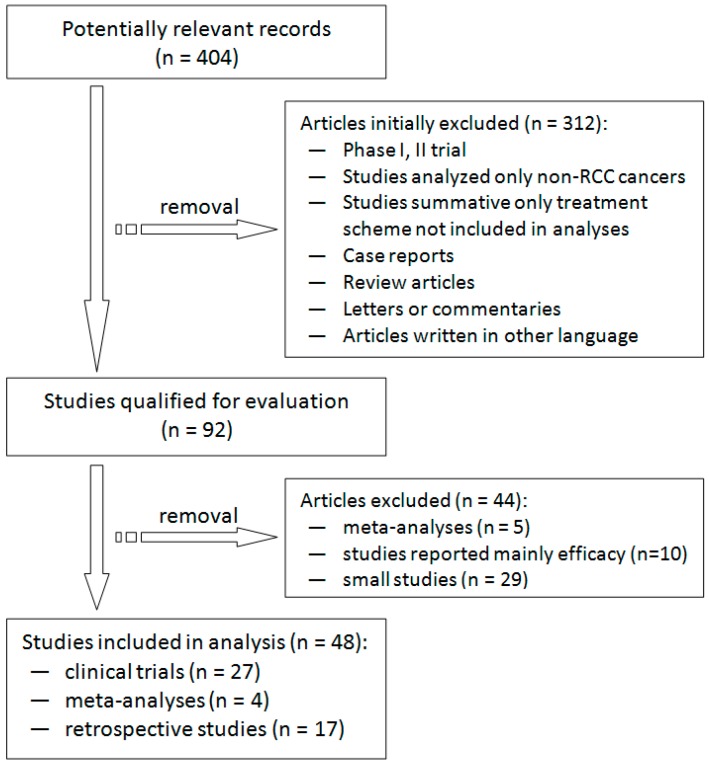
Flow chart of the literature selection process.

We identified 404 potentially relevant published articles. According to the criteria described in the “Selection criteria” section, a total of 48 articles were included in the systematic review. There were 27 clinical trials, 4 meta-analyses and 17 retrospective studies (Figure 1). Toxic side effects in reviewed articles were graded according to the Common Terminology Criteria for Adverse Events (CTCAE) version 3.0 if not specified otherwise and are summarized in Table 1 [4].

## 3. Discussion

### 3.1. Hypertension

Hypertension is a common side effect of anti-VEGF therapies. Hypertension of any grade according to CTCAE was reported with an incidence ranging from 17% to 49.6% in the reviewed randomized controlled trials (Table 2) [5,6,7,8,9,10,11,12,13,14,15,16,17,18,19,20,21,22].

Unlike previous reports, in the PREDICT study (Patient characteristics in REnal cell carcinoma and Daily practICe Treatment with sorafenib), hypertension was detected in 4.2% of patients treated with sorafenib; however, it was a non-interventional study, with no additional diagnostic or monitoring procedures beyond standard local clinical practice [22]. Four meta-analyses of phase 2, 3 and 4 clinical trials conducted in various types of cancer found an incidence of all grade hypertension of 25.9% for sunitinib, 38.2% for pazopanib, 24.9% for sorafenib and 57.6% for axitinib, and high grade hypertension of 8.3% for sunitinib, 6.8% for pazopanib, 8.6% for sorafenib and 26.1% for axitinib, respectively, in the subgroup of mRCC patients [23,24,25,26]. In the Qi et al study, the frequency of all-grade hypertension associated with pazopanib was notably higher than that of sorafenib and sunitinib, whereas the incidence of high-grade hypertension linked with pazopanib was comparable to that of sorafenib and sunitinib, and, in the Hall et al. study, sorafenib was connected with the largest risk of the development of hypertension [24,27]. The risk of developing all grade hypertension with axitinib was also significantly higher than with sunitinib and sorafenib, while the risk of high grade hypertension with axitinib was higher than with other VEGFR-TKIs [26]. Differences in the incidence of hypertension among VEGFR-TKIs may stem from the distinctive pharmacodynamic effects. The in vitro half maximal inhibitory concentration (IC50) for axitinib against VEGFR 1–3 was 10-fold lower compared to other VEGFR-TKIs [28]. Lorenzo et al. reported that only 17.6% of patients with sunitinib induced grade 3 hypertension were normotensive before treatment with sunitinib [29]. Matrana et al. described 43% of patients treated with pazopanib as having had exacerbations of pre-existing hypertension during therapy, and only 6% patients developed new-onset hypertension [30]. In the study by Hamnvik et al., risk factors of VEGF treatment-induced hypertension included pre-existing hypertension, age above 60 years and BMI above 25 kg/m^2^ [31]. Maitland et al., however, detected that the magnitude of sorafenib-induced blood pressure (BP) elevation varies with individuals, and the BP variability is not associated with the baseline blood pressure nor the variability in total plasma concentrations of the drug [32]. In a study by Kim et al., the likelihood of sunitinib-induced hypertension was connected with *VEGF* Single Nucleotide Polymorphisms (SNPs). The authors detected a substantial affiliation between the prevalence of hypertension and the *VEGF* SNP −634 genotype, as patients with the less advantageous GG genotype were appraised to have roughly 13- to 14-fold greater likelihood of being hypertensive during therapy compared with patients with the CC genotype [33]. In a study by Eechoute et al., a greater increase in systolic blood pressure during the first sunitinib treatment cycle was associated with the presence of an ACG haplotype in *VEGFA:* rs699947 (−2578 A > C), rs833061 (−460 C > T), and rs2010963 (405 C > G). The grade 3 hypertension was significantly associated with the presence of an ACG haplotype in *VEGFA* and the presence of a C allele in *eNOS* rs2070744 (−786 T > C) [34]. Diekstra et al. reported that sunitinib-induced hypertension was associated with the presence of the T allele in *IL-8* rs1126647. There is some evidence that IL-8, by upregulating VEGF levels, can play a role in stimulating VEGFR-2 transactivation [35]. Van Erp et al. revealed that the development of hypertension was related to the *VEGFR-2* 1191CT and TT genotypes [36]. Quin et al. showed that patients with the rs1045642 CT + TT variant in *ABCB1*, which is connected with sorafenib pharmacokinetics, led to an increased risk of treatment-related hypertension in the Chinese population [37]. However, Noda et al. reported that total sunitinib concentration was not correlated with the severity of hypertension [38]. Sunitinib-induced hypertension may be related to SNPs in cytochrome *P450 3A4* (*CYP3A4*) that transform sunitinib -to its active metabolite. Diekstra et al. observed that A-allele carriers of *CYP3A4* rs4646437 had a higher incidence of hypertension compared with wild type (WT) carriers of *CYP3A4* [39]. Investigators also detected an association between SNPs and blood pressure changes during axitinib treatment. Patients with the *VEGFR-2* rs2305948 C/T genotype had elevated diastolic blood pressure more frequently [40]. Polymorphisms in *ABCB1* that are connected with sorafenib pharmacokinetics may result in individual changes in drug absorption in the small intestine. Thus, they may be associated with the differences in toxicity. Similarly, patients with the *CYP3A4* rs4646437 genotype probably have increased exposure to the drug with stronger inhibition of the VEGF pathway. Table 3 summarizes the SNPs that are associated with a higher risk of the development of hypertension in patients treated with TKI.

Blood pressure elevation induced by sunitinib or sorafenib was detectable within the first few days of treatment [32,41]. During sunitinib treatment in 175 patients, grade 3 hypertension was reported after the first and second cycles in 1.71% of patients, 4% of patients developed hypertension after cycle 3, while 2.3%, 1.14% and 0.6% of patients developed hypertension after cycles 4, 5 and 6, respectively [29]. Similarly, the median time to grade ≥3 axitinib-induced hypertension was three months and the rate of all grade hypertension in patients receiving axitinib declined during the two years of treatment [41]. Porta et al. reported that the incidence of all grade hypertension in patients treated with sunitinib decreased from 34% in the first year to 29% in the second year of therapy and then remained relatively stable [42]. In analyses performed by Kaymakcalan et al., hypertension led to dose modification in 1% of patients treated with VEGF-targeted therapies in routine practice [43].

**Figure 2:**
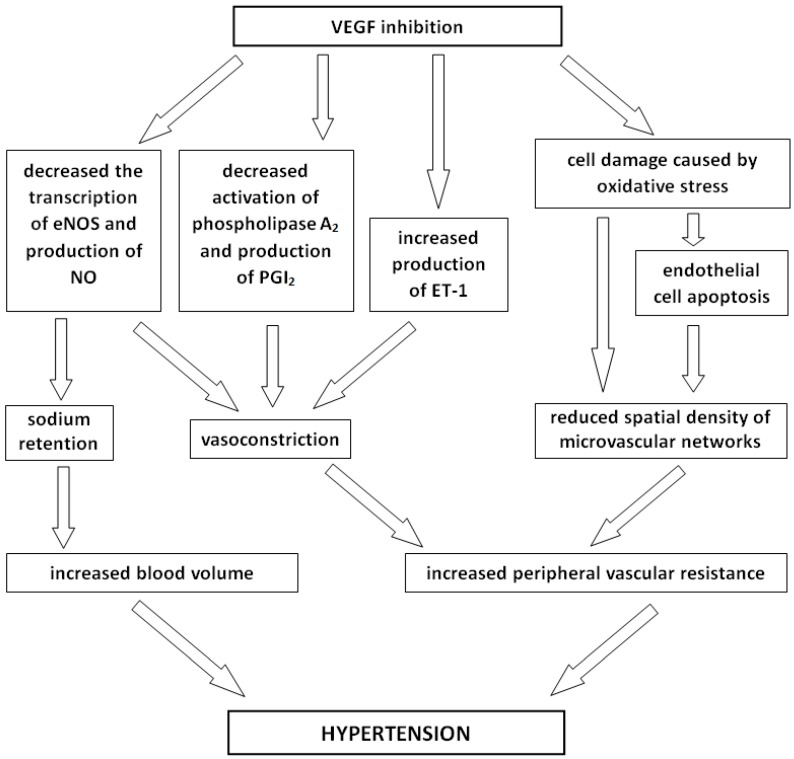
The pathogenesis of hypertension in patients receiving the vascular endothelial growth factor signaling pathway inhibitor (anti-VEGF) therapy. VEGF inhibition leads to decreased transcription of endothelial nitric oxide synthase (eNOS), decreased activation of phospholipase A_2_, and increased production of the endothelin-1 (ET-1). Decreased production of nitric oxide (NO), increased production of prostacyclin (PGI_2_), and the endothelin-1 (ET-1) resulting in vasoconstriction. The inhibition of VEGF leads to endothelial cell damage and, thereafter, to a reduction in microvascular density. The vasoconstriction and rarefaction resulting from an increase in vascular resistance. Anti-VEGF treatment also results in increased blood volume via sodium retention caused by the decreased NO production. An increased vascular resistance and an increased blood volume are directly related to hypertension development.

The pathogenesis of hypertension in patients receiving anti-VEGF therapy likely relates to multiple pathways and is not yet fully understood. Emerging evidence implicates increased peripheral vascular resistance in the pathophysiology of anti-angiogenic therapy-induced hypertension, and proposed mechanisms include reduced formation of nitric oxide by endothelial cells, an increased production of vasoconstrictive factors, and a reduction in microvascular density (rarefaction). VEGF promotes the transcription of endothelial nitric oxide synthase (eNOS), thus increasing the production of nitric oxide (NO), and also induces the production of prostacyclin (PGI_2_) via the activation of phospholipase A_2_, resulting in vasodilation [46,47]. Hence, decreased NO and PGI_2_ production, resulting from the inhibition of VEGF, may lead to vasoconstriction and increased blood pressure. Another proposed mechanism of increased peripheral resistance includes heightened activation of the endothelin-1 (ET-1), a potent vasoconstrictor, which was detected in plasma in higher concentrations during sunitinib treatment [48]. Experimental evidence that VEGF–induced hypertension is caused by the dysregulated production of vasodilators and vasoconstrictors is somewhat contradictory. Van der Veldt et al. showed that sunitinib treatment is not associated with impaired microvascular endothelium-dependent and endothelium-independent vasodilatation, while Thijs et al. reported that the development of hypertension precedes the reduction of endothelium-dependent vasodilation in patients treated with sunitinib [49,50]. Another proposed mechanism of VEGF blockade-associated hypertension includes a process called rarefaction, which is defined as a reduced spatial density of microvascular networks. VEGF plays an important role in the proliferation of new blood vessels and takes part in maintaining endothelial cell viability and structure. Thus, the inhibition of VEGF leads to endothelial cell apoptosis and remodeling of the capillary beds in many tissues resulting from an increase in vascular resistance [51,52]. Investigators found that capillary density decreased during anti-VEGF therapy and is directly related to an increase in blood pressure [49]. Other mechanisms have also been evaluated as potential contributors to anti-angiogenic therapy-induced hypertension. VEGF is one of the factors that protects endothelial cells against damage secondary to oxidative stress. Thus, VEGF inhibition caused by the administration of angiogenesis blockers may be one of the causes of hypertension [53]. Anti-VEGF treatment also results in sodium retention via decreased NO production [54]. Investigators determined that sunitinib treatment was also associated with a fall in plasma renin concentration and plasma renin activity, whereas plasma concentrations of aldosterone did not change; therefore, the possibility that mineralocorticoid-receptor activation plays a role in the development of hypertension cannot be excluded [48]. The presumable mechanisms of hypertension in patients receiving anti-VEGF therapy are summarized in Figure 2.

### 3.2. Proteinuria

In addition to hypertension, proteinuria is a common side effect attributable to the anti-angiogenic agents, and a direct marker of the nephrotoxicity of the therapy. The incidence and rate of proteinuria are variable in different studies according to patient characteristics and targeted signals. Table 1 and Table 2 summarize the National Cancer Institute’s proteinuria grading and findings of the available phase III and IV studies concerning the proteinuria induced by VEGF-targeted therapies.

Baek et al. reported that proteinuria developed in 17.6% of patients, and preexisting proteinuria was aggravated in 23.1% of patients after the initiation of sunitinib therapy [55]. Symptomatic nephrotic syndrome developed in 0.9% of patients. Among the patients with proteinuria, 80.9% discontinued the treatment, after which proteinuria improved in 70% of patients and persisted in 30%. Investigators found that proteinuria occurred in 11.64% patients treated with axitinib in the first-line and in 20.74% of patients treated with axitinib in the second-line. Similarly, proteinuria was detected in 12.50% of patients treated with sorafenib in the first-line and in 20.29% of patients treated with sorafenib in the second-line [56]. In the study by Miyake et al., 41.5% of Japanese patients treated with axitinib developed proteinuria [57]. The authors also detected that the proportion of patients with hypertension in the proteinuria group was significantly higher than in the group without proteinuria. Despite the high incidence, most cases of proteinuria are asymptomatic and not severe, with nephrotic range proteinuria (>3.5 g/day) occurring in 1%–5% of patients depending on the trial (Table 2).

In the COMPARZ (Pazopanib Versus Sunitynib in the Treatment of Locally Advanced and/or Metastatic Renal Cell Carcinoma) study, which compared the efficacy and safety of pazopanib and sunitinib as first-line therapy, proteinuria led to the discontinuation of treatment in 3% of patients treated with pazopanib and 1% of patients treated with sunitinib [21]. In analyses performed by Sorich et al., the median time to any grade proteinuria in patients treated with pazopanib or sunitinib was 32 days and to grade 3/4 proteinuria was 100 days [58]. In the study by Baek et al., proteinuria developed at a mean of 163 days after the initiation of sunitinib treatment; risk factors were hypertension, dyslipidemia, and chronic kidney disease at the initiation of sunitinib therapy [55]. In the multivariable analysis, patients with preexisting proteinuria, higher systolic blood pressure (SBP), and Asian ethnicity had significantly higher risk of proteinuria connected with treatment [58]. The authors also observed that nephrectomy was connected with a reduced risk of proteinuria. Patients with preexisting grade 1 proteinuria had an 8.1% risk of grade 3/4 proteinuria, and patients without preexisting proteinuria had a 2.7% risk of grade 3/4 proteinuria. Similarly, diabetes was a statistically significant risk factor. Patients treated with sunitynib had a trend towards a reduced risk of proteinuria compared to patients treated with pazopanib; however, no influential difference in the risk of grade 3/4 proteinuria was ascertained between drugs. Land et al. conducted a retrospective review of patients with metastatic renal cell cancer treated with pazopanib and showed that 80% of patients developed proteinuria during 12 months of treatment [59]. All patients experienced a grade 1 or 2 proteinuria and the time to resolution of proteinuria after the end of treatment was up to 17 months. Investigators showed a higher incidence of proteinuria than others, which may be connected with comorbid disease state relating to hypertension and diabetes. The authors reported that 78% of patients had a comorbid disease at baseline, but these were not assessed in the study. At the same time, 80% of patients had no baseline proteinuria.

Proteinuria may also be developed by axitinib treatment. In the study by Nozawa et al., 22% of patients developed a proteinuria score of 4+ during treatment [60]. Rini et al. reported that the rate of all grade proteinuria during axitinib treatment increased from 11% to 19% over time, and the rates of grade 3 proteinuria stabilized over time [41].

The pathogenesis of proteinuria in patients receiving anti-VEGF therapy is still unknown and likely relates to multiple pathways. VEGF is essential to the maintenance of renal function, and interruption in the expression of VEGF may disorganize normal glomerular function. Few patients who developed proteinuria while on therapy underwent renal biopsies. The most common pathological findings were thrombotic microangiopathy (TMA), which is indicative of vascular damage [61]. Other observed results include crescentic glomerulonephritis, focal segmental glomerulosclerosis lesions (FSGS), immune complex-mediated glomerulonephritis minimal change nephropathy (MCN), and acute interstitial nephritis [62,63,64,65,66]. The performed biopsies also revealed acute tubular injury and tubular necrosis [61,66]. The relative ampleness of VEGF was reduced in podocytes from patients with MCN and FSGS and was undetectable in the kidney with TMA when compared with control tissues [67]. The interaction between VEGF generated by podocytes and VEGFR-2 on glomerular endothelial cells helps to sustain glomerular vascular permeability [68]. A loss of endothelial fenestrations in the capillaries, proliferation of glomerular endothelial cells (endotheliosis) and loss of podocytes is caused by VEGF inhibition [51,68]. Anti-VEGF treatment also leads to a decrease in the expression of nephrin, subsequently resulting in podocyte injury and proteinuria. The presumable mechanisms of proteinuria in patients receiving anti-VEGF therapy are summarized in Table 4. VEGFR-2 interacts in vitro and in vivo with nephrin via the *Akt* transduction pathway, which is upregulated after nephrectomy in the remaining kidney as part of a compensatory mechanism [69]. There is some evidence that a central role in podocyte dysfunction leading to proteinuria is played by the protein c-mip, which interferes with the nephrin/Akt signaling pathway [70]. The protein c-mip may decrease the phosphorylation of nephrin and cause cytoskeletal disorganization, thereby leading to the dysfunction of the slit diaphragm [71]. The relative abundance of c-mip was greatly increased upon anti-VEGF treatment and was associated with the subsequent development of FSGS and MCN [70]. Anti-VEGF therapy is also associated with low *RelA* expression that binds to the *c-mip* promoter in vivo and in vitro and represses its transcription. Increased RelA levels, which is a subunit of NF-κB, was detected in TMA kidneys [71]. Echeverria et al. reported that sorafenib inhibited NF-κB activation [72]. This disturbance leads to c-mip overexpression and may indirectly induce podocyte disease with proteinuria [71].

Another proposed mechanism of proteinuria would include paraneoplastic nephropathies or the concurrent presence of unrelated renal disease. RCC patients may develop IgA nephropathy, FSGS and membranous nephropathy as a paraneoplastic syndrome [73,74].

### 3.3. Renal Function

Treatment with anti-VEGF was also associated with an increased risk of renal dysfunction. Motzer et al. reported that sunitinib was associated with an all-grade creatinine increase in 70% of patients and grade 3/4 events reported in less than 1% of patients; in the study by Vrdoljak et al., increased blood creatinine was detected in 5.2% of patients [5,9]. Kidney function deterioration was detected in 6.35% of patients treated with axitinib and in 5.21% of patients treated with sorafenib during the first-line treatment; the frequency of renal dysfunction during the second-line was similar [56]. In meta-analyses performed by Zhu et al., the incidence of all-grade creatinine increase was 65.6% in the patients receiving sunitinib [23]. Rini et al. announced that one treatment-related death due to increased blood creatinine and c-reactive protein (CRP) was reported in the sorafenib group of the AXIS (Comparative effectiveness of axitinib versus sorafenib in advanced renal cell carcinoma) study [17]. Patients treated with sunitinib had a higher risk of kidney function deterioration than patients treated with pazopanib [21]. Sunitinib treatment in Asian patients was connected with a decreased estimated glomerular filtration rate (eGFR). The median relative change in eGFR during treatment was 21% and patients taking sunitinib for a long time tended to have a greater depletion in the eGFR [75]. In the study by Beak et al., kidney function deterioration occurred in 7.7% of patients, and the maximum creatinine level in the course of therapy was 3.31 mg/dL [55]. The average time from the commencement of the treatment to the deterioration of renal function was 199 days. All patients in whom kidney function declined stopped taking the medication, but kidney function improved only in 16.6% of patients. In the performed analyses, older patients were significantly more exposed to kidney injury during therapy. Khan et al. reported that kidney function began to worsen in a median of 2.1 months (range 0.4–19.1) following the start of sunitinib or sorafenib treatment, and patients with chronic kidney disease at the start of treatment had a longer interval to maximum kidney function deterioration compared with patients with normal renal function (6.6 versus 4.6 months); however, the difference was not statistically significant [76]. Qin et al. found that 39.2% of patients treated with axitinib and 35.4% of patients treated with sunitinib had increased creatinine levels [20]. Similarly, in the study by Miyake et al., in the Japanese population, renal function tended to worsen during anti-VEGF treatment; however, no significant differences in the reduction in eGFR were detected according to the CKD-EPI (Chronic Kidney Disease-Epidemiology Collaboration) formula during sunitinib-, sorafenib- or axitinib-treatment [77]. Treatment duration was significantly related to a reduction in eGFR >10% across all lines of targeted therapy. In the study by Khan et al., patients treated with sunitinib experienced a median increase in serum creatinine of 0.8 mg/dL (range 0.3–2.8) and a median decrease in eGFR of 25 mL/min (range 8.54–64.76) [76]. Rini et al. reported that the rate of blood creatinine level during axitinib treatment increased from 3% to 7% over time [41].

The mechanism of renal dysfunction during VEGF-TKI treatment has not been fully understood. The loss of VEGF function through pharmacologic inhibition was associated with damage to glomerular endothelial cells and podocytes. Additionally, anti-VEGF treatment leads to vasoconstriction via decreased NO and PGI_2_ production and results in decreased blood flow in the glomeruli.

Kidney function deterioration is also linked with nephrectomy or contrast-induced nephropathy (CIN) following contrast-enhanced computed tomography (CT). The loss of nephrons during partial or radical nephrectomy can predispose patients to chronic renal failure. Chung et al. showed that the incidence of new-onset CKD in stage G3 (eGFR 30–59 mL/min/1.73 m^2^) and G4 (eGFR 15–29 mL/min/1.73 m^2^) after radical nephrectomy (RN) was 36.1% and 3.4%, respectively [78]. They also noticed a significant drop in the eGFR calculated using the MDRD (Modification of Diet in Renal Disease Study) equation in the first three months after nephrectomy; however, kidney function gradually increased after a nadir eGFR value was reached. The authors detected a successive improvement over the following 60 months in patients with preoperative eGFR above 15 mL/min/1.73 m^2^. Small studies that have examined the association between type of surgery and renal functional outcomes demonstrated that partial nephrectomy results in a significantly lower risk of renal dysfunction than radical unilateral nephrectomy [79,80,81]. In the study by Mason et al., the median eGFR in the CKD-EPI equation was 19.6 mL/min/1.73 m^2^ lower in patients undergoing unilateral radical nephrectomy [82]. A lower preoperative eGFR, increasing age, larger tumor size and hypertension were associated with a lower postoperative eGFR at 24 months postoperatively. Launay-Vacher et al. analyzed kidney function in patients with kidney cancer after unilateral nephrectomy and those treated with any anti-angiogenic therapy and showed that the eGFR decreased during treatment in all patients; however, the mean decline in GFR was similar to physiological reduction in renal function [83]. A greater decrease in renal function was observed only in a subgroup of patients with hypertension at baseline (decrease in eGFR of 26.23 to 30.31 mL/min/1.73 m^2^/year depending on the assessment method used).

Contrast media administration was linked to acute kidney injury (AKI); however, McDonald et al. found that the rate of AKI was similar between contrast recipients and control groups in patients with baseline eGFR higher than 30 mL/min/1.73 m^2^, providing evidence that CIN may not be a clinical concern in these patients [84]. These results were confirmed by Kim et al., who showed that no patient with an eGFR above 45 mL/min/1.73 m^2^ developed CIN [85]. Baseline kidney function deterioration was connected with an increased risk of CIN. The frequency of contrast-induced nephropathy in patients with eGFR of 30–45 mL/min/1.73 m^2^ was 2.9%, and in patients with eGFR <30 mL/min/1.73 m^2^ was 12.1%.

Renal side effects and hypertension in mRCC patients are related to multiple mechanisms; however, one of the main disturbances in those patients is VEGF inhibition connected with TKI treatment. There is some evidence that these side effects may be used as biomarkers of response to anti-angiogenic agents. A number of antecedent studies have evidenced an interdependence between the occurrence of hypertension in patients receiving sunitinib or axitinib and outcomes [86,87,88]. In addition, associations between certain VEGF and VEGFR-2 SNPs with hypertension and clinical benefits have been found [32]. Rini et al. reported that systolic blood pressure (BP) ≥140 mmHg and diastolic BP ≥90 mmHg in patients treated with sunitinib were associated with notably better results than patients with lower systolic and diastolic BP (median overall survival of 30.5 months vs. 7.8 months and 32.1 months vs. 15 months, respectively); however, Goldstein et al. detected that for week 4 and week 12, there were no significant associations between progression-free survival (PFS) and SBP increase in patients treated with sunitinib or pazopanib [89,90]. The potential clinical benefits suggest that patients with hypertension linked with anti-VEGF treatment should continue the treatment of malignancies and start to take antihypertensive medicines. There are no special recommendations for the treatment and those patients should be treated in agreement with standard guidelines [2,54]. Baek et al. showed that the median PFS for patients in whom proteinuria developed or was aggravated during sunitinib treatment was significantly longer (median PFS, 245 days, 95% CI 150 to 340 vs. median PFS, 469 days, 95% CI 198 to 740, *p* = 0.020) [55]. There is evidence that kidney function deterioration may also be associated with improved PFS [75]. These results suggest that the detection of renal side effects or hypertension should not lead to treatment discontinuation, especially if the severity of the adverse events is mild. According to current knowledge, there is no recommendations of treatment after the occurrence of grade 4 nephrotoxicity following TKI treatment. One of the options that should be considered is starting treatment with mTOR inhibitors that target signaling pathways other than TKI. The other is the continuation of TKI treatment despite renal toxicity; however, studies in these fields are also expected.

## 4. Methods

### 4.1. Search Strategy

Studies were searched for among PubMed databases using the following terms and strategy: (“anti-angiogenic drugs” or “TKI” or “sorafenib” or “sunitinib” or “pazopanib” or “axitinib”) and (“renal cell cancer” or “RCC” or “metastatic renal cell cancer”) and (“kidney injury” or “proteinuria” or “hypertension” or “AKI”) in the abstract or in the title. The databases were searched for studies published up to June 2016. We also examined the clinical trial registration website [56] and the references in the analyzed articles in order to obtain additional information. Disagreements were resolved by discussion between the two reviewers.

### 4.2. Selection Criteria

Studies were defined as eligible if they (1) were written in English, presenting data from phase 3 or 4 clinical trials as well from retrospective clinical studies; (2) included patients of any sex aged ≥18 years with mRCC; (3) reported adverse events (AEs) with or without reporting on efficacy either in the first- or subsequent-line settings; (4) reported adequate AE data or data allowing such outcomes to be computed; and (5) were published as original articles (no case reports, case series, reviews, comments, letters, or editorials). The decision to include or exclude studies was hierarchical, originally based on the study title, followed by the abstract, and finally the complete body text. We included in the analysis only the most recent data, studies with the longer follow-up, or the most relevant studies if several articles were based on the same patient material. In addition, in the case of the accessibility of large studies reporting AEs that we considered, data relating to 100 patients or less and confirming results were omitted. We excluded studies that examined the effect of neoadjuvant or adjuvant treatment. Combined modality designs, i.e., sunitinib combined with any standard or experimental agent, were also excluded.

## 5. Conclusions

Anti-VEGF drugs represent effective treatment options for patients with mRCC but are associated with renal toxicity. These effects are most often mild in severity but may sometimes lead to treatment discontinuation. There are limited data on the incidence and mechanism of the renal-related toxicities caused by targeted agents; however, our report highlights the importance of blood pressure and kidney function monitoring during TKI therapy, especially in elderly patients and patients with a prior history of hypertension and/or kidney disease. Most of the information regarding nephrotoxicity of VEGF inhibitors comes from retrospective trials in which information was collected only for clinical practice reasons and depends on the quality of the data input by individual physicians. To compare safety profiles of all available kinase inhibitors, head-to-head trials based on patients with the same entry level characteristics are required. The mechanism of renal dysfunction during a TKI treatment has also not been fully understood, so further research to reveal the basic mechanisms of renal effects of these agents is warranted. It might be useful to include biomarkers of kidney damage in addition to conventional parameters. These data are important with respect to patient care, given the importance of balancing anti-VEGF treatment-associated toxicities with the benefits obtained by treating malignancies.

## Figures and Tables

**Table 1:** Side effects graded according to the Common Terminology Criteria for Adverse Events (CTCAE) version 3.0 [5].

Adverse Event	Grade				
	1	2	3	4	5
Hypertension	Asymptomatic, transient (<24 h) increase by >20 mmHg (diastolic) or to >150/100 if previously WNL; intervention not indicated	Recurrent or persistent (≥24 h) or symptomatic increase by >20 mmHg (diastolic) or to >150/100 if previously WNL; monotherapy may be indicated	Requiring more than one drug or more intensive therapy than previously	Life-threatening consequences (e.g., hypertensive crisis)	Death
Proteinuria	1+ or 0.15–1.0 g/24 h	2+ to 3+ or >1.0–3.5 g/24 h	4+ or >3.5 g/24 h	Nephrotic syndrome	Death
Creatinine increased	>ULN–1.5 × ULN	>1.5–3.0 × ULN	>3.0–6.0 × ULN	>6.0 × ULN	Death

WNL: Within Normal Limits; ULN: Upper Limit of Normal.

**Table 2:** Incidence of the tyrosine-kinase inhibitors (TKI) targeted therapy-associated hypertension and proteinuria in phase III/IV clinical trials.

Reference	Author	Treatment	Number of Patients	Hypertension		Proteinuria	
				All Grade (%)	≥3 (%)	All Grade (%)	≥3 Grade (%)
[5]	Motzer et al., 2009	sunitinib	375	112 (30)	45 (12)	-	-
[6]	Gore et al., 2015	sunitinib	4543	1104 (24)	267 (6)	-	-
[7]	Akaza et al., 2015	sunitinib	1671	584 (35)	168 (10)	-	-
[8]	Sternberg et al., 2014	sunitynib	521	135(26)	27 (5)	-	-
[9]	Vrdoljak et al., 2015	sunitynib	401	93 (23)	28 (7)	-	-
[10]	Sternberg et al., 2013	pazopanib	290	116 (40)	13 (4)	30 (10)	7 (3)
[11]	Escudier et al. 2007	sorafenib	451	76 (17)	16 (4)	-	-
[12]	Procopio et al. 2007	sorafenib	136	36 (26)	2 (1.4)	-	-
[13]	Beck et al., 2011	sorafenib	1145	223 (19.5)	70 (6.1)	-	-
[14]	Motzer et al., 2013	sorafenib	257	88 (34)	46 (18)	187 (73)	7 (3)
[15]	Motzer et al., 2014	sorafenib	286	79 (28)	47 (17)	-	-
[16]	Akaza et al., 2015 *	sorafenib	3255	1171 (36)	65 (2)	-	-
[17]	Rini et al., 2011	axitynib	359	145 (40)	56 (16)	-	-
		sorafenib	355	103 (29)	39 (11)	-	-
[18]	Hutson et al., 2013	axitynib	189	92 (49)	26 (13)	-	-
[19]	Motzer et al., 2013	sorafenib	355	107 (30)	43 (12)	27 (8)	4 (1)
		axitynib	359	149 (42)	60 (17)	45 (13)	11 (3)
[20]	Qin et al., 2015	axitynib	135	67 (49.6)	26 (19.3)	28 (20.7)	7 (5.2)
[21]	Motzer et al., 2013	pazopanib	554	257 (46)	82 (15)	98 (18)	23 (4)
		sunitynib	548	223 (41)	81(15)	75 (14)	22 (4)
[22]	Jäger et al., 2015	sorafenib	2599	114 (4.2)	-	-	-

* Adverse events (AEs) were summarized based on the medical dictionary for regulatory activities (MedDRA), version 15.0 terminology, and classified into serious and non-serious according to the seriousness criteria defined in International Conference on Harmonization Guideline E2A [44,45].

**Table 3:** Single Nucleotide Polymorphisms associated with higher risk of development of hypertension.

Reference	Single Nucleotide Polymorphisms	Full Name of Gene	VEGF Inhibitor
[30]	VEGF rs2010963 (−634 G > C)	vascular endothelial growth factor	sunitinib
[31]	VEGFA rs699947(−2578 A > C)	vascular endothelial growth factor A	sunitinib
	VEGFA rs833061 (−460 C > T)		
	VEGFA rs2010963 (405 C > G)		
[33]	*VEGFR-2* rs2305948 (1191 C > T)	vascular endothelial growth factor receptor 2	sunitinib
[37]	VEGFR-2 rs2305948 (1192 C > T)	vascular endothelial growth factor receptor 2	axitinib
[31]	IL-8 rs1126647 (A > T)	interleukin 8	sunitinib
[31]	eNOS rs2070744 (−786 T > C)	nitric oxide synthase	sunitinib
[34]	ABCB1 rs1045642 (C > T)	ATP binding cassette subfamily B member 1	sorafenib
[36]	CYP3A4 rs4646437 (G > A)	cytochrome P450 family 3 subfamily A member 4	sunitinib

**Table 4:** The causes of proteinuria in patients receiving the vascular endothelial growth factor signaling pathway inhibitor (anti-VEGF) therapy.

The Causes of Proteinuria in Patients Receiving Anti-VEGF Therapy	
The slit diaphragm dysfunction	loss of endothelial fenestrations in the glomeruli
	endothelial cells cytoplasm swelling
	podocyte damage
	decreased expression of nephrin
The narrowing or occlusion of capillary lumina by basement membrane	
Acute interstitial nephritis	
Acute tubular necrosis	
